# Gender-based differences in host behavior and gut microbiota composition in response to high fat diet and stress in a mouse model

**DOI:** 10.1038/s41598-017-11069-4

**Published:** 2017-09-07

**Authors:** Laura C. Bridgewater, Chenhong Zhang, Yanqiu Wu, Weiwei Hu, Qianpeng Zhang, Jing Wang, Shengtian Li, Liping Zhao

**Affiliations:** 1 Department of Microbiology and Molecular Biology, Brigham Young University, Provo, Utah USA; 2 State Key Laboratory of Microbial Metabolism, School of Life Sciences and Biotechnology, Shanghai Jiao Tong University, Shanghai, P.R. China; 3 Bio-X Institutes, Shanghai Jiao Tong University, Shanghai, P.R. China

## Abstract

Obesity is associated with a high prevalence of mood disorders such as anxiety and depression. Both stress and high fat diet can alter the gut microbiota and contribute to obesity. To examine the interrelationships between obesity, stress, gut microbiota and mood disorders, obesity was induced in mice using a high fat diet, and the mice were subsequently stressed using a chronic unpredictable mild stress protocol. During the experiment, the composition of the gut microbiota was analyzed by 16 S rRNA gene high-throughput sequencing, and anxiety-like behaviors were measured. The results revealed distinct gender differences in the impacts of obesity and stress on anxiety-like behaviors, activity levels, and composition of the gut microbiota. Male mice were more vulnerable to the anxiogenic effects of the high fat diet, and obese male mice showed decreased locomotion activity in response to stress whereas obese female mice did not. In females, stress caused the gut microbiota of lean mice to more closely resemble that of obese mice. Taken together, these results suggest the importance of considering gender as a biological variable in studies on the role of gut microbiota in obesity-related mood disorders.

## Introduction

Converging lines of evidence indicate that a dysbiotic gut microbiota contributes to obesity and its associated metabolic phenotypes including heart disease, insulin resistance, fatty liver, and systemic inflammation^1^. For example, transplanting gut microbiota from obese mice or humans increased fat deposits in recipient germ free mice^2–4^. Antibiotic treatment to alter the gut microbiota reduced obesity, insulin resistance, and systemic inflammation in high fat diet-fed mice^5^. In humans, transplanting gut microbiota from lean donors improved peripheral and hepatic insulin sensitivity in obese recipients^6^.

Obesity, in turn, is associated with a high prevalence of mood disorders such as anxiety and depression^7, 8^. The association of mood disorders with obesity has been replicated in several rodent models, and proposed pathophysiological mechanisms for the connection include excessive production of proinflammatory cytokines that indirectly affect serotonin production^9^, elevated leptin levels acting on leptin receptors in the brain^10^, neuroinflammation as an extension of the systemic inflammation that commonly accompanies obesity^11^, and high fat diet-induced alterations in glucocorticoid signaling in the hippocampus^12^. Adipose tissue can secrete proinflammatory cytokines and leptin, providing a route by which adipose tissue can directly modulate neurological function, but transplantation of gut microbiota from obese donor mice on a high fat diet (HFD) produced behavioral changes in recipient mice even though the recipients did not develop obesity, suggesting that the gut microbiota can also directly modulate some of the neurological complications of obesity^13^.

Stress, like a HFD, can alter the composition of the gut microbiota^14–18^. Stress can also trigger mood disorders like anxiety and depression^19, 20^, and it can promote obesity^21^, suggesting that the impact of stress on mood disorders and obesity might be at least partially attributable to the changes it causes in the gut microbiota.

To examine the interrelationship between diet, stress, gut microbiota, and mood disorders, we used a high fat diet (HFD) to alter the gut microbiota and induce obesity in mice and then measured anxiety-like behavior. We subsequently stressed the mice using a chronic unpredictable mild stress protocol and once again examined both anxiety-like behavior and the gut microbiota. These studies demonstrated gender differences in the impact of HFD and stress on anxiety-like behaviors and locomotor activity.

## Materials and Methods

### Animals

**Figure 1:**
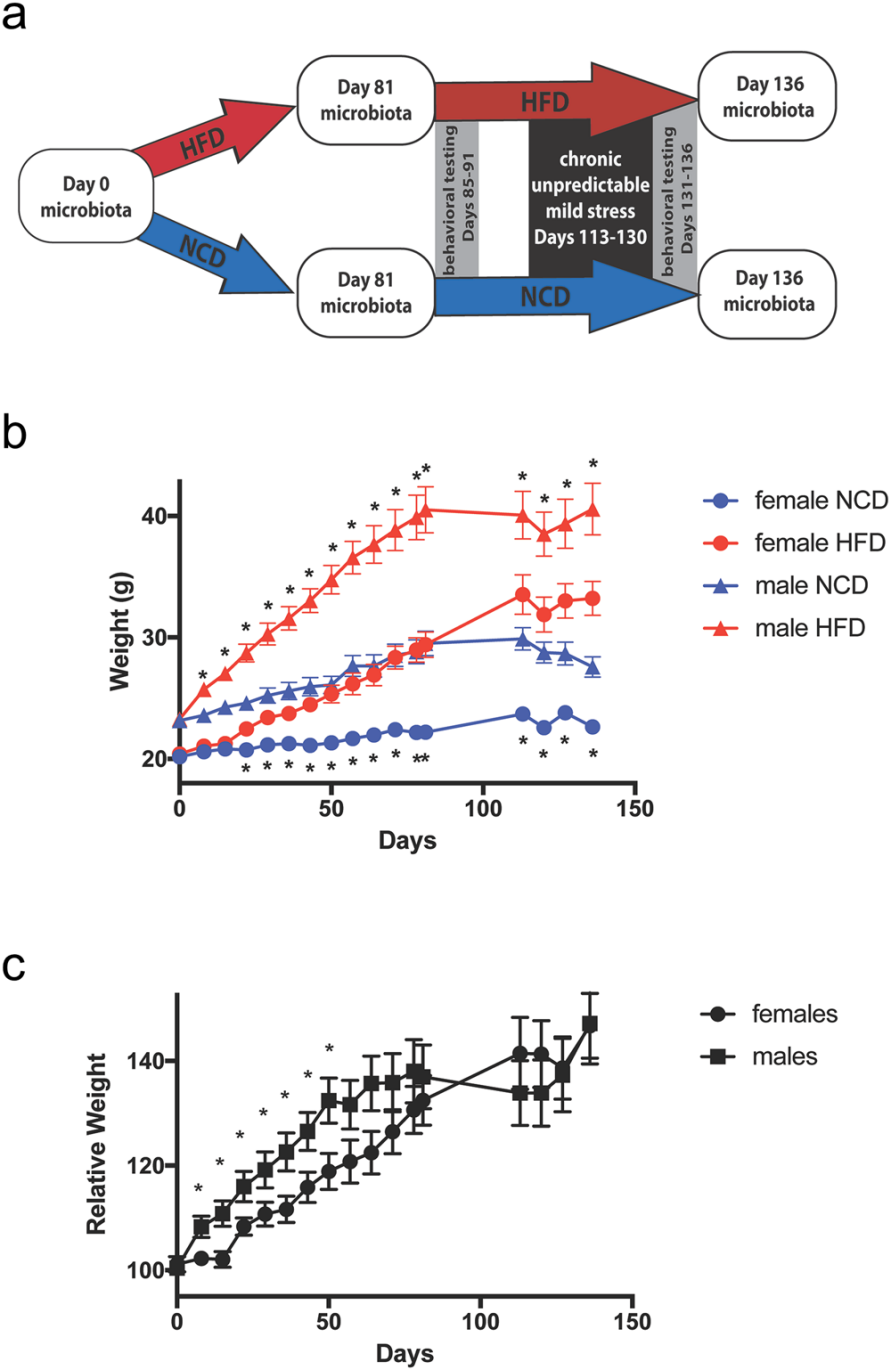
Schematic overview and weight tracking. (**a**) Overview of the experiment design showing microbiota sample collection (white boxes), dietary groups (red and blue arrows), behavioral testing (grey boxes) and stress (back box). HFD, high fat diet; NCD, normal chow diet. (**b**) Weight in grams of female and male mice on the NCD and HFD, showing Mean ± standard error (SE). Stars above the lines indicate significant differences between NCD and HFD in males, and stars below the lines indicated significant differences between NCD and HFD in females (p < 0.01). (**c**) Relative body weight in female and male mice (Mean ± SE), calculated as the weight of each HFD mouse normalized to the average weight of NCD-fed mice of the same gender at the same time point. All time points that showed significant differences between males and females are marked (p < 0.05). No significant differences were detected after Day 50. N = 14–17 per group.

6-week old C57BL/6 mice were purchased from SLAC Inc. (Shanghai, China). All animals were shipped at the same time and housed, 4 to a cage, in the same room of the SFP rodent facility. Mice were and acclimated in the facility on a 12hr/12hr light/dark cycle for 2 weeks on a normal chow diet (NCD) containing 10% of total energy from fat, 70% from carbohydrate, and 20% from protein (Research Diets D12450B, New Brunswick, New Jersey, Supplementary Figure 1). All animals were shipped at the same time and housed in the same room within the same rodent facility building. At 8 weeks of age, baseline body weight was measured and fecal samples were collected from all animals for microbiome analysis (day 0, baseline), and then half of the female and half of the male mice were switched to a high fat diet (HFD) containing 60% of total energy from fat, 20% from carbohydrate, and 20% from protein (Research Diets D12492, Supplementary Figure 1). (See Fig. 1a for a schematic overview of research design.) Animals remained on the same diets for the duration of the study. Body weight was measured weekly. Fecal samples were collected for microbiota analysis at day 81 (after the first 12 weeks on HFD) and at day 136 (after stress). During days 85–91 and days 131–136, mice were subject to behavioral testing as described below. During days 113–130, mice were subject to chronic unpredictable mild stress as described below. All operations and experimental methods were approved by the Animal Care and Use Committee of the School of Life Sciences and Biotechnology, Shanghai Jiao Tong University (No. 2014–005), and carried out according to its guidelines.

### Behavioral testing

Animals were brought to the behavioral testing room at least one hour before the beginning of testing for acclimatization. The order of testing alternated between groups to control for any time-of-day-specific effects. All tests were performed during the animals’ light cycle.

#### Open field test

Mice were placed in the center of a square open field arena, 80 cm × 80 cm × 25 cm, and allowed to explore for 5 min. Movements were recorded digitally, and then tracked and analyzed using ANY-maze video tracking software (Stoelting Co., Wood Dale, Illinois). The arena was divided into a 4 × 4 grid, and the percent of total distance traveled in the central four squares of the grid (25% of the arena) was quantified. Decreased percent of distance in the central portion of the arena was interpreted as increased anxiety-like behavior^22^.

#### Elevated plus maze

Mice were placed in the center of the elevated plus maze facing an open arm and allowed to explore the maze for 5 min. Movements were recorded digitally, tracked, and analyzed using Mobiledatum Animal Behavior Analysis System application software v2.0. Time spent in the open arms and percent of arm entries into open arms were measured, and decreased time spent in open arms or decreased entries into open arms was interpreted as increased anxiety-like behavior^23^. Total distance traveled and the number of entries into all arms were also quantified as measures of locomotion.

#### Other behavioral testing

Mice were also subject to the forced swim test and the tail suspension test in an attempt to measure despair/depression-like behavior^24, 25^, but these tests were inconclusive due to tail climbing behavior in the tail suspension test and decreased fur quality in HFD-fed mice, which decreased buoyancy in the forced swim test. These tests did, however, contribute additional stress to the mice, extending the period of stress caused by the chronic unpredictable mild stress protocol. The order of testing was open field test, elevated plus maze, forced swim test, tail suspension test.

### Chronic unpredictable mild stress

Mice were subject to chronic unpredictable mild stress for 18 days before the second series of behavioral testing began. Stressors included 1 or 2 episodes per day of the following, administered in random order: damp bedding for 18 hrs; forced swim for 5 min in cold (8 °C) water; tilting animal in a cage without bedding back and forth 18 times at a 45° angle each direction; lights on during the dark cycle; lights off during the light cycle; predator smells; predator sounds; and forced swim for 5 min in hot (40 °C) water.

### Gut microbiota profiling

Microbial DNA was extracted as described by Godon *et al*.^26^ from fecal samples collected at days 0, 81, and 136. The V3-V4 region of 16S rRNA gene was amplified, and DNA libraries were prepared from 192 samples (n = 17 for NCD females, HFD females, and HFD males at days 0 and 81; n = 16 for NCD males at days 0 and 81; n = 15 for NCD females and HFD males at day 136; n = 14 for HFD females and NCD males at day 136) according to Illumina’s directions (http://res.illumina.com/documents/products/appnotes/16s-metagenomic- library-prep-guide.pdf), and were sequenced using the Illumina Miseq platform.

### Analysis of sequencing data

Both the forward and reverse ends of the same read were truncated at the first base where the Q value was no more than 2. If the pair of reads had a minimum overlap of 50 bp, they were merged into a complete read. These reads were not kept unless longer than 399 bp with an expected error of no more than 0.5^27^. Quality-filtered reads were dereplicated into unique sequences and then sorted by decreasing abundance, and singletons were discarded. Representative non-chimeric OTU sequences were next picked by Uparse’s default^27^. Further reference-based chimera detection was performed using UCHIME against the RDP Classifier training database (v9). The OTU table was finalized by mapping quality-filtered reads to the remaining OTUs with the Usearch global alignment algorithm at a 97% cut off. Representative sequences for each OTU were built into a phylogenetic tree by FastTree and subjected to the RDP Classifier to determine the phylogeny with a bootstrap cut off of 80% (RDP database 16 S rRNA training set 14)^28, 29^.

The samples were removed from further analysis if the high-quality reads of samples were less than 4600 (Supplementary Table 1), and then the sequences of all the samples were downsized to 4600 (1000 permutations) to equalize the difference in sequencing depth. All subsequent analysis was performed based on the QIIME platform (version 1.8)^30^. The alpha-diversity of each sample was calculated with observed OTUs, Faith’s phylogenetic diversity (PD Whole tree) and the Shannon index. Principal coordinate analysis and MANOVA were then performed based on the Bray-Curtis distances^31^.

### Statistical analyses

Random Forest models were introduced to identify specific bacterial phylotypes that contributed to the segregation of gut microbiota by sex, diet or stress^32^. The samples from all the females and males at Day 0 were used to establish classification models of sex. The classification models of diet or stress were established for females and males separately. Samples from mice on the NCD and HFD on Day 0 and Day 81 were used to establish classification models of diet. Samples from mice on Day 81 and Day 136 on NCD or HFD were used to establish classification models of stress. Each Random Forest model was run with 1,000 trees and leave-one-out cross validation for improved generalization error. The importance of an OTU was determined based on the mean decrease in accuracy of discrimination.

The correlation among OTUs was calculated using the SparCC algorithm with a bootstrap procedure repeated 999 times. The Ward clustering algorithm and PERMANOVA (9999 permutations, *P* < 0.001) based on SparCC correlation coefficients were used to cluster the OTUs into co-abundance groups (CAGs) using the R program.

Body weights were analyzed at each time point using unpaired Student’s t-test. Behavioral testing results were analyzed for significance using two-way ANOVA with an alpha of 0.05.

### Accession numbers

The raw Illumina read data for all samples has been deposited in the Sequence Read Archive (SRA) under the accession number SRP091784.

## Results

### The high fat diet produced similar relative weight gain in female and male mice

When male mice were placed on the high fat diet (HFD), they became significantly heavier than normal chow diet (NCD) mice within one week (Fig. 1b). In contrast, females did not show a significant difference until three weeks after initiation of the HFD (Fig. 1b). In order to compare weight gain between male and female mice, relative weight was calculated by dividing each HFD mouse’s weight by the average of all NCD mice of the same gender at that time point. During the first 7 weeks, males showed significantly greater relative body weight than females at each time point, possibly due to the 2-week delay in weight gain when female mice were first placed on the HFD. From the 7-week time point onward, there were no significant differences in relative body weight between males and females (Fig. 1c), making it unlikely that any of the differences in locomotor activity between males and females detected at the 12-week or 18-week time points were attributable to gender-specific differences in weight gain.

### Male mice showed more behavioral sensitivity to the high fat diet than did females

**Figure 2:**
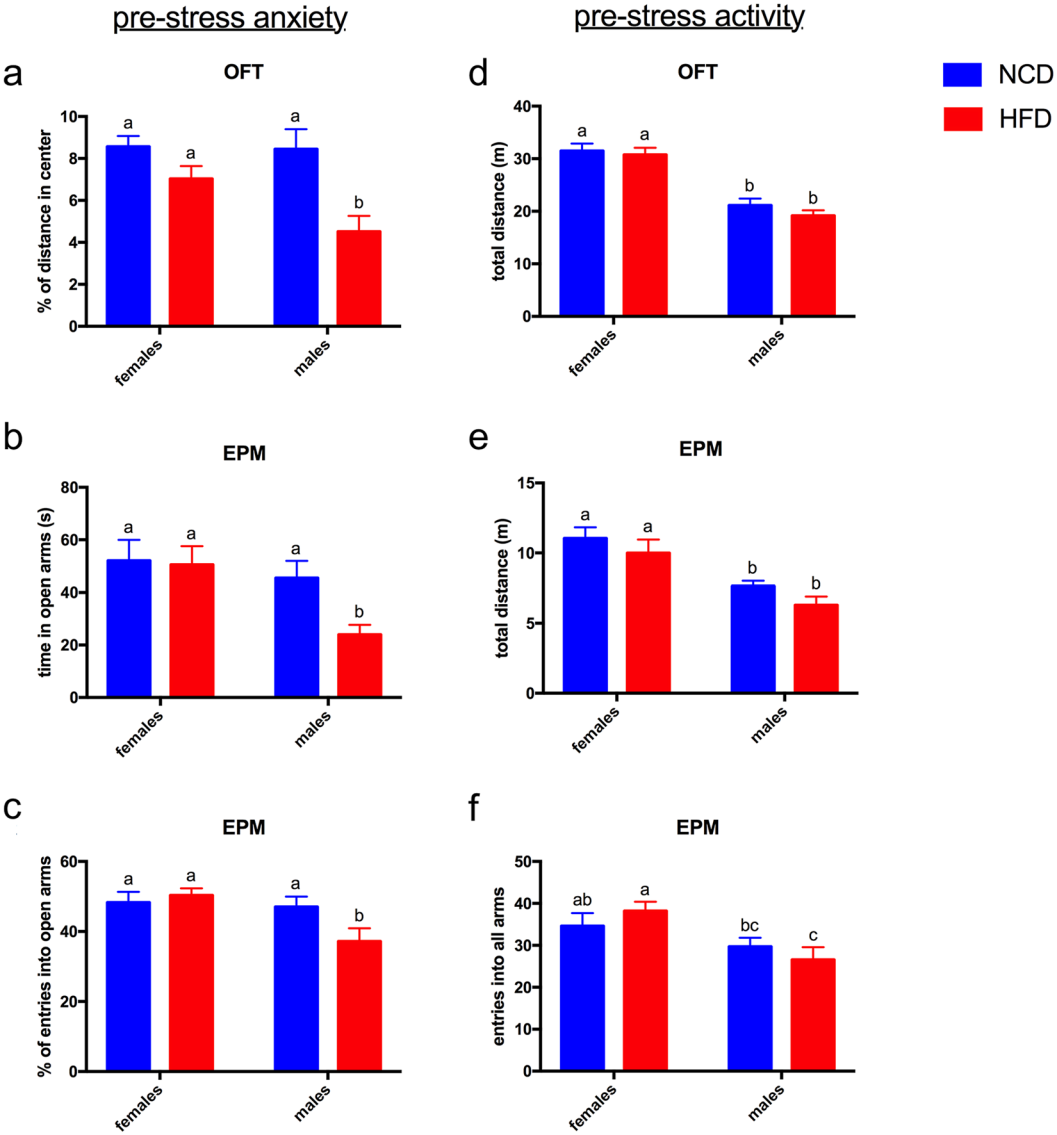
Male but not female mice show increased anxiety-like behaviors in response to high fat diet. (**a**) Percent of the total distance traveled that was spent in the central 25% of the arena in the open field test (OFT). (**b**) Seconds spent in open arms in the elevated plus maze (EPM). (**c**) Percent of total entries into open (as opposed to walled) arms in the EPM. (**d**–**f**) Male mice showed less activity than females on both diets as measured by (**d**) total distance traveled in the OFT, (**c**) total distance traveled in the EPM, and (**e**) number of entries into all arms in the EPM. Diet had no impact on activity levels in male or female mice. All graphs show Mean ± SE. Letters above bars indicate statistical significance (p < 0.05)—bars with the same letter are not significantly different from each other. N = 14–17 per group.

After 12 weeks on the HFD, each mouse was placed in the open field test arena and allowed to explore for 5 min. Its path was tracked, and the distance traveled in the inner 25% of the arena was calculated as a percentage of the total distance traveled. Decreased percentage of distance in the center indicates increased anxiety^22^. Male mice on the HFD showed a significant decrease in center distance compared to male mice on NCD as well as to female mice on HFD (Fig. 2a).

Anxiety-like behavior was also measured in the elevated plus maze, where decreased time in and/or number of entries into the open arms indicate increased anxiety^23^. Again, male mice on the HFD showed significantly more anxiety-like behavior than male mice on the NCD or female mice on either diet (Fig. 2b and c). Female mice on the HFD showed no increase in anxiety-like behaviors compared to females on the NCD in either the open field test or the elevated plus maze (Fig. 2a,b,c). Male mice, therefore, appeared to be more sensitive to the anxiogenic impact of the HFD than were females.

Locomotor activity was assessed by measuring total distance traveled in the open field test and the elevated plus maze, and total number of entries into all arms in the elevated plus maze. By all three measures, males showed less locomotor activity than females. Diet had no impact on locomotor activity in males or females (Fig. 2d,e,f).

### Male mice on the HFD showed decreased locomotor activity in response to stress

**Figure 3:**
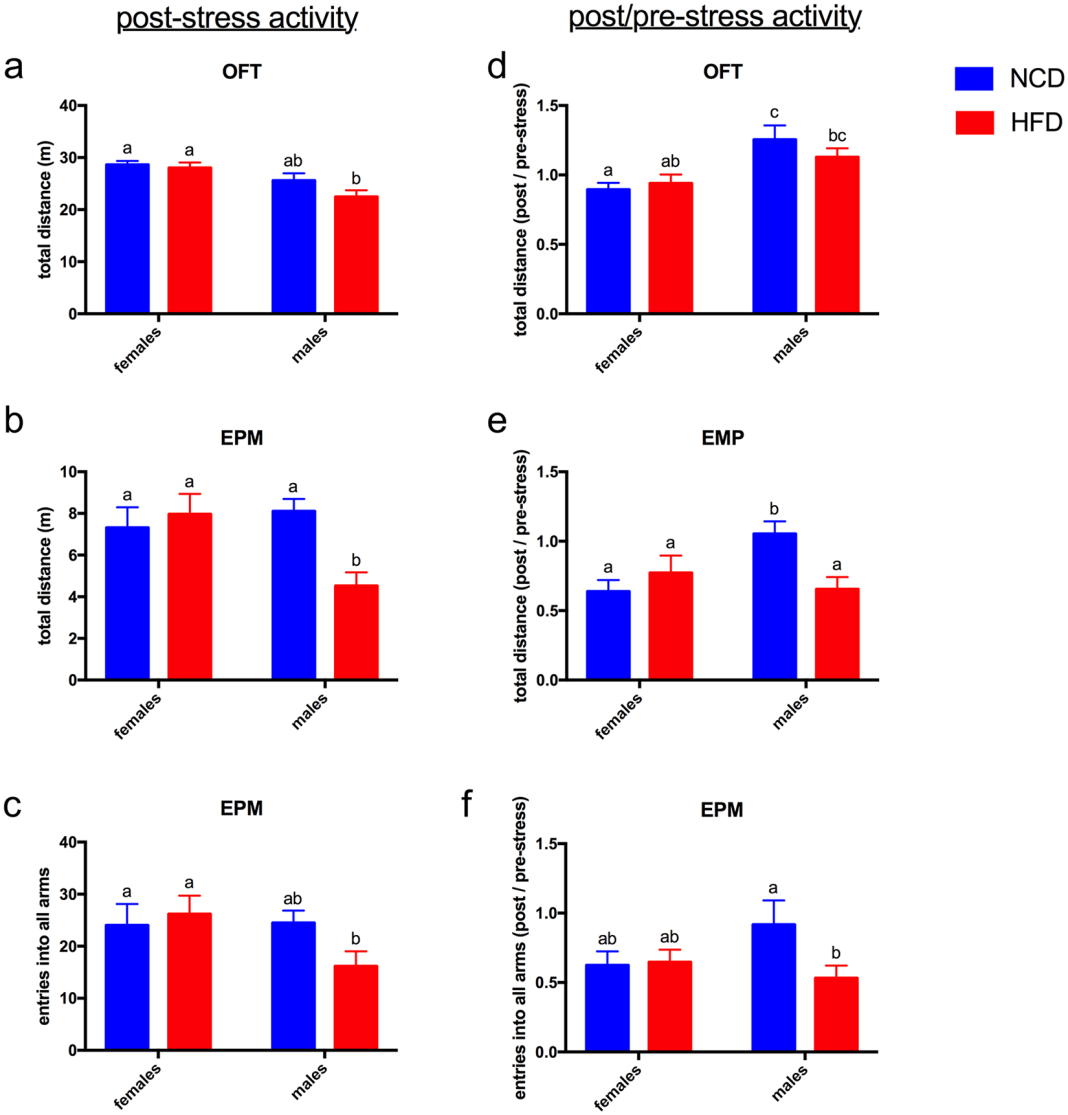
Diet affects the impact of stress on activity in male mice. (**a**–**c**) After stress, males on HFD showed reduced locomotion activity compared to females on either diet. Males on NCD were not different from females, and the activity of females on the two diets was not different from each other. (**d**–**f**) In order to normalize for any differences that might be caused by performing the same behavioral tests twice in the same mice (separated by 7 weeks), post-stress results were divided by pre-stress results to obtain a ratio that was then compared between female and male mice and between mice on the different diets. The ratio of post/pre-stress activity was significantly less in males on HFD compared to NCD in the elevated plus maze (EPM). All graphs show Mean ± SE. Letters above bars indicate statistical significance (p < 0.05)—bars with the same letter are not significantly different from each other. N = 14–17 per group.

The elevated plus maze and the open field test were repeated in the same mice following a 2 ½ week period of chronic unpredictable mild stress, 7 weeks after the initial round of behavioral testing (Fig. 3, left panels). To eliminate the confounding effects of reduced novelty during the second exposure to the test arenas, results were calculated as a ratio of post-stress to pre-stress measurements, and differences in the degree of change between groups were compared statistically (Fig. 3, right panels).

The post/pre-stress ratios showed no gender- or diet-specific differences in anxiety-like behaviors after stress (data not shown). Activity measures, however, did show differences. In raw data measurements of locomotor activity after stress, males on HFD showed decreased locomotion compared to females (Fig. 3a,b,c) and compared to NCD males (Fig. 3b). After normalization of post-stress to pre-stress data, HFD males showed a significantly greater stress-related reduction in locomotion than did NCD males (Fig. 3e,f). Females on both diets also showed a significantly greater stress-related reduction in locomotion than did NCD males (Fig. 3d,e). Diet had no effect on females’ behavioral responses to stress (Fig. 3a–f).

### Diet, gender, and stress were all associated with differences in the mouse gut microbiota

Gut microbiota was sampled at Day 0 (baseline), Day 81 (after 12 weeks on the HFD), and Day 136 (after chronic unpredictable mild stress) and analyzed by 16S rRNA gene V3-V4 region sequencing on an Illumina MiSeq platform. After quality-control filtering, we obtained 1,396,744 reads from 190 samples with an average of 7,351 reads per sample (±1,907 standard deviation), and 351 operational taxonomic units (OTUs) were delineated with a 97% cutoff (Supplementary Table 1).

**Figure 4:**
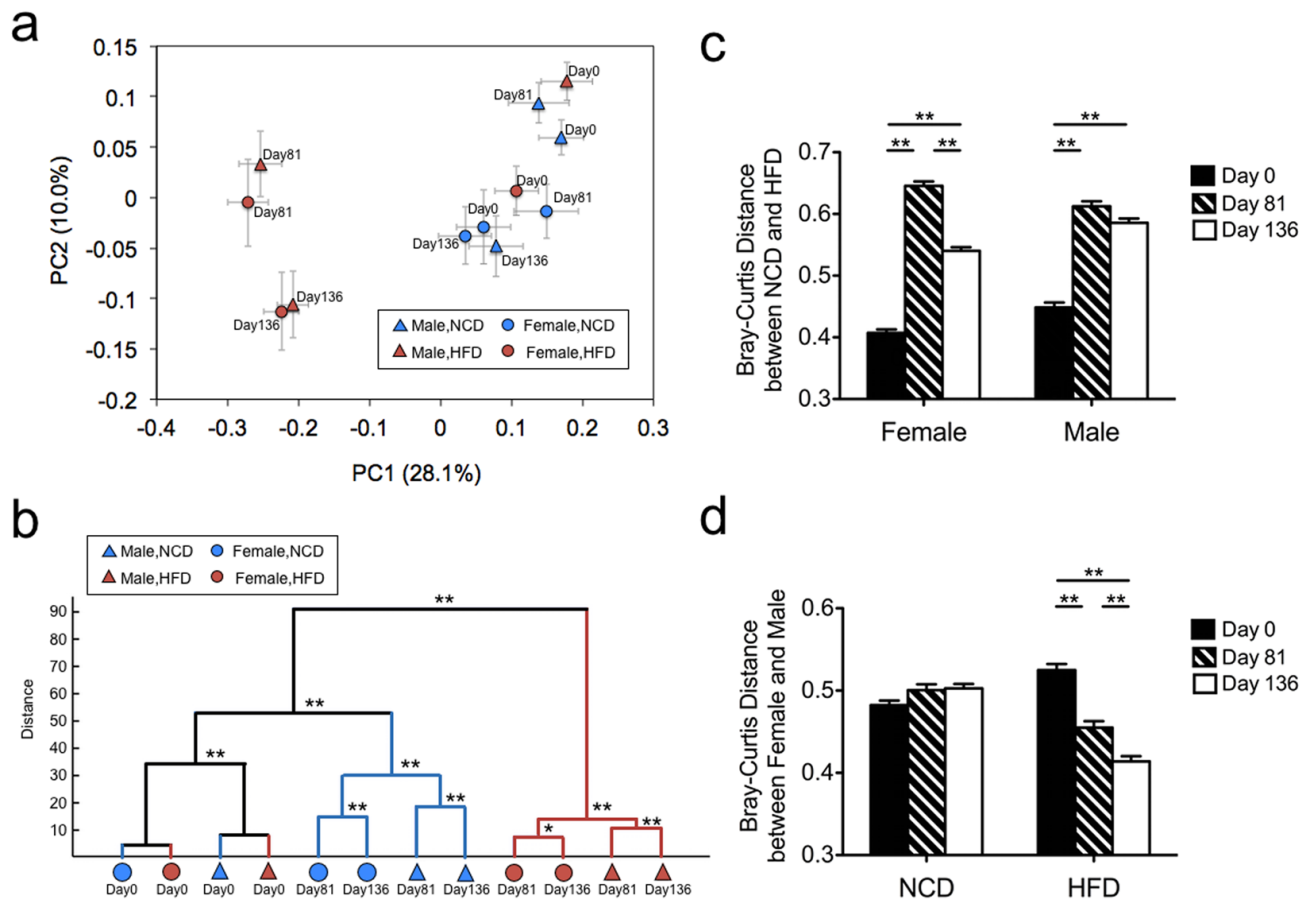
Beta diversity analysis reveals that diet, gender, and stress significantly alter the composition of the gut microbiota. (**a**) Principle coordinate analysis (PCoA) based on Bray-Curtis dissimilarity of all OTUs from all the groups on Day 0, 81 and 136. (**b**) Clustering of gut microbiota based on distance between different groups calculated by MANOVA testing of the first 20 principal coordinates (accounting for 80% of total variation). (**c**) Comparison of the Bray-Curtis distance between NCD and HFD at each time point. (**d**) Comparison of the Bray-Curtis distance between females and males at each time point. All data is showed as mean ± SE For (**c** and **d**) the tests of significance were performed using a two-sided Student’s t-test with Bonferroni-correction, **P < 0.01 and *P < 0.05. N = 13–17 per group.

HFD produced a dramatic shift in the composition of the gut microbiota as indicated by principle coordinate analysis (PCoA) based on Bray-Curtis distance of all OTUs (Fig. 4a). Clustering of gut microbiota based on distances between the different groups calculated by MANOVA testing of the first 20 principle coordinates (accounting for 80% of total variation) shows groups separating first by diet, then by gender, then by stress (Fig. 4b).

Comparison of the Bray-Curtis distances between NCD and HFD revealed that stress decreased the distance between the gut microbiota of NCD and HFD mice in females but not in males (compare Day 81 to Day 136, Fig. 4c). A similar comparison between female and male mice revealed that when mice were on NCD, the distance between their gut microbiota remained steady, even after stress (Fig. 4d). In mice on HFD, in contrast, distance between male and female gut microbiota decreased with time on the HFD, and stress further reduced the distance (Fig. 4d).

### Female and male gut microbiota were different at Day 0

**Figure 5:**
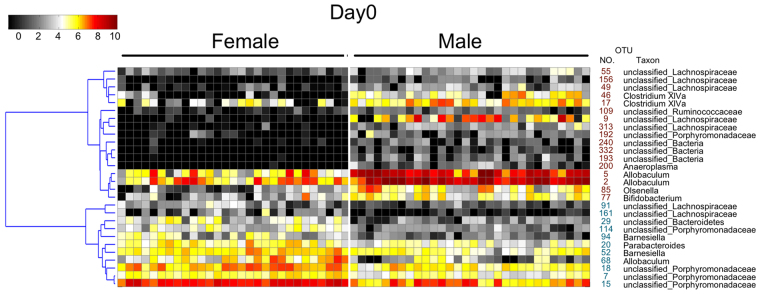
Key phylotypes of gut microbiota showed differences between female and male mice. Key phylotypes were identified as those OTUs with a value of discrimination mean decrease in accuracy greater than 0.002 in Random Forest models of female and male mice on Day 0. The heat map shows the abundance (log 2 transformation) of OTUs. OTU identification numbers are shown along the right side of the heat map, with red print indicating greater abundance in male mice and blue indicating greater abundance in female mice. Hierarchical clustering of the OTUs was performed using the Spearman rank correlation distance metric.

The diversity and structure of gut microbiota showed significant difference between female and male mice before diet and stress treatment (Supplementary Figure 2 and Fig. 4). The OTUs that were significantly different between males and females on Day 0 were identified using Random Forest modeling (estimated error of leave-one-out cross validation was 0, Supplementary Figure 3). Seventeen OTUs were significantly greater in male mice, and 11 OTUs were significantly greater in female mice (Fig. 5). Because of these striking differences, all subsequent analyses were performed separately in males and females.

### HFD produced marked shifts in the gut microbiota

**Figure 6:**
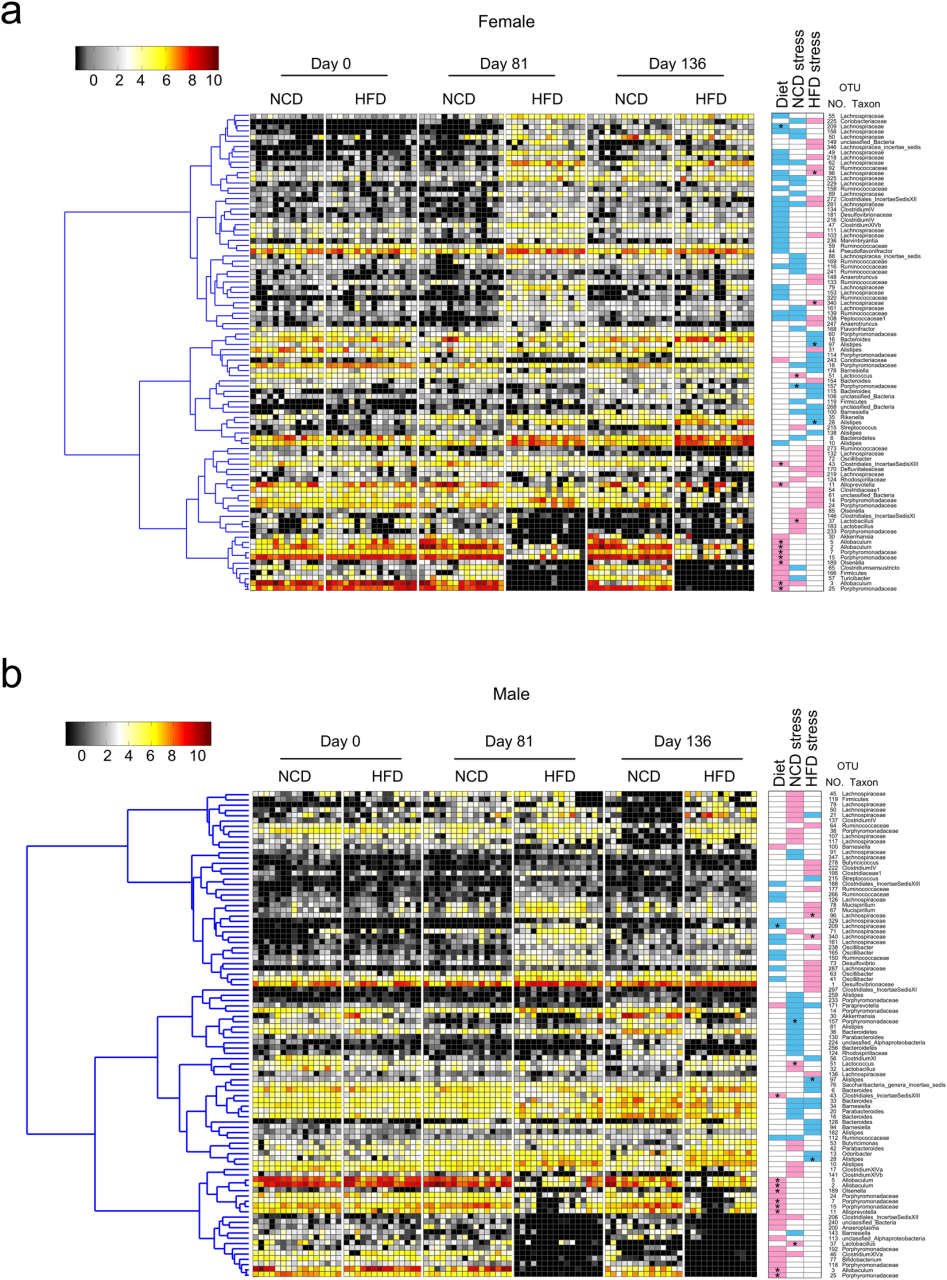
Stress in females shifted the NCD gut microbiota to resemble the HFD gut microbiota. A combined analysis was performed using Random Forest models to identify all OTUs that changed significantly in response to either diet or stress in (**a**) female and (**b**) male mice. OTUs are arranged according to their co-occurrence clusters based on Spearmann correlation coefficients (left side of heat map). The key comparisons and direction of changes are summarized in three columns at right as follows: “Diet” compares NCD mice at Day 81 to HFD mice at Day 81; a blue box indicates that the OTU was higher with HDF, and a pink box indicates that it was lower with HFD. “NCD stress” compares NCD mice at Day 81 (pre-stress) to NCD mice at Day 136 (post-stress); a blue box indicates that the OTU was higher after stress, and a pink box indicates that it was lower after stress. “HFD stress” compared HFD mice at Day 81 (pre-stress) to HFD mice at Day 136 (post-stress); a blue box indicate that the OTU was higher after stress, and a pink box indicates that it was lower after stress. A star means that the OTU responded similarly in female and male mice.

The OTUs that were significantly different between NCD and HFD on day 81 were identified by Random Forest models (estimated error of leave-one-out cross validation for female was 0.034, for male was 0). Twenty-seven OTUs increased and 14 decreased in females in response to HFD (Fig. 6a, Supplementary Figure 4A). Twelve increased and 20 decreased in males in response to HFD (Fig. 6b, Supplementary Figure 4B). OTUs that changed similarly in male and female mice are marked by stars in Fig. 6a and b. Nine of these OTUs decreased in both males and females, while only one (OTU 209, which belongs in Lachnospiraceae) increased in response to HFD in both males and females. No OTUs changed in opposite directions in males and females.

### Stress shifted the gut microbiota in the absence of HFD

The OTUs that shifted in response to chronic unpredictable mild stress were identified using Random Forest models to compare Day 81 to Day 136 in females and in males on NCD (estimated error of leave-one-out cross validation for female was 0.1, for male was 0.1). Twenty-three OTUs were increased by stress in females, and 10 OTUs were decreased (Fig. 6a and Supplementary Figure 4C). Interestingly, the list of stress-increased OTUs included 7 of the same OTUs that were increased by HFD alone in females, including 4 in Lachnospiraceae, 2 in Ruminococcaceae, and 1 in Peptococcaceae. One OTU in *Allobaculum* was decreased by both stress and HFD in females (Fig. 6a).

In males, 20 OTUs were increased by stress and 20 were decreased (Fig. 6b and Supplementary Figure 4D). Of these OTUs, only one (in Ruminococcaceae) was also increased by HFD and two (in Clostridiales Incertae Sedis XII and in *Clostridium* XIVa) were also decreased by HFD (Fig. 6b).

### Stress affected the gut microbiota differently if mice were on HFD rather than NCD

The effects of stress on the microbiota of mice on the HFD was examined using Random Forest modeling to compare Day 81 to Day 136 in females and in males on HFD (estimated error of leave-one-out cross validation for female was 0.071, for male was 0.27). In females on HFD, stress caused 15 OTUs to increase and 26 OTUs to decrease (Fig. 6a and Supplementary Figure 4E). In males on HFD, stress caused 14 OTUs to increase and 17 OTUs to decrease (Fig. 6a and Supplementary Figure 4F). Comparison of the OTU changes induced by stress in mice on NCD versus HFD revealed that only 4 OTUs in females and 3 OTUs in males were changed similarly by stress in NCD and HFD mice, suggesting that the microbiota’s response to stress is powerfully shaped by diet.

### Stress in females shifted the NCD gut microbiota to resemble the HFD microbiota

When the effects of diet, stress, and gender were analyzed together, the most striking aspect of the resulting heat maps was the similarity between NCD and HFD in female mice after stress (Day 136), compared to their dissimilarity before stress (Day 81) (Fig. 6a, and also supported by Fig. 4c). Stress in female NCD mice caused the gut microbiota to more closely resemble the microbiota produced by long-term consumption of HFD, even though the female NCD mice showed no weight gain. Male mice did not show the same effect (Fig. 6b), demonstrating gender-specific differences in the way the gut microbiota responds to HFD and to stress.

### OTUs that responded together to diet and stress can be clustered into co-abundance groups

**Figure 7:**
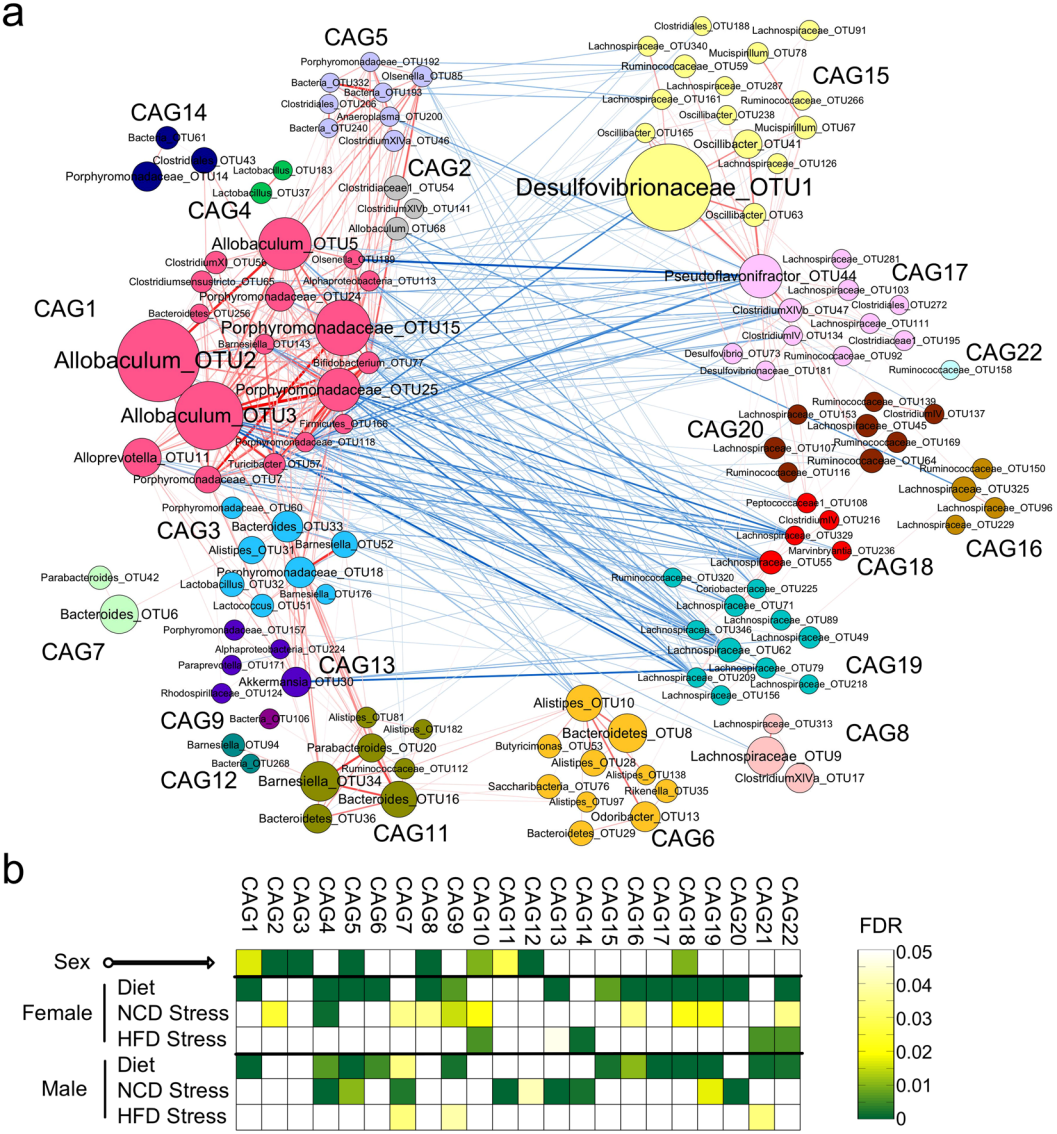
Co-abundance groups of the all the key OTUs in mice. (**a**) Network plot highlighting correlation relationships between 20 CAGs of prevalent OTUs (162 OTUs selected by Random Forest modeling). Node size indicates the average abundance (Hellinger tansformed) of the OTUs. Lines between nodes represent SparCC correlations between the nodes they connect, with line width indicating the correlation magnitude, and red and blue colors indicating positive and negative correlations, respectively. For clarity, only lines corresponding to correlations whose magnitude is greater than 0.4 were drawn, and unconnected nodes were omitted. (**b**) Heatmap of the false discovery rate (FDR) for CAGs, indicating the changes induced by sex, diet or stress. The tests of significance were performed using a two-sided Mann-Whitney test, and the calculation of FDR was performed using the procedure originally introduced by Benjamini and Hochberg^33^.

Correlation relationships between the most prevalent OTUs (shared by greater than 30% of the samples at all time points) revealed 22 co-abundance groups (CAGs) (Fig. 7a and Supplementary Figure 5). Correlations of the abundance of each CAG with sex, diet, and stress were performed using a two-sided Mann-Whitney test, and a calculation of the false discovery rate (FDR) was performed using the procedure originally introduced by Benjamini and Hochberg^33^. Nine of the CAGs were significantly different between females and males at Day 0 indicating gender-specific differences (Fig. 7b, top row of squares). HFD had a strong impact on 16 of the CAGs, with 11 of those 16 CAGs affected similarly by HFD in both females and males, 3 affected only in females, and 2 affected only in males (Fig. 7b, “Diet” rows).

Unlike HFD, which produced largely similar changes in CAGs between females and males, stress affected mostly different CAGs in females and males. Seven CAGs were shifted by stress in females only, 6 CAGs were shifted by stress in males only, and 3 CAGs were shifted by stress in both females and males (Fig. 7b, “NCD Stress” rows). Comparing the effects of HFD to stress revealed that in females, 7 of the same CAGs were shifted by HFD alone and by stress alone, consistent with our observation (Fig. 6a) that stress in females caused the NCD gut microbiota to more closely resemble the HFD gut microbiota. In males, 4 of the same CAGs were shifted by HFD alone and by stress alone.

When the effects of HFD and stress together were examined by subjecting mice on the HFD to stress, few additional changes in CAG abundance were observed beyond what the HFD alone generated. Females that received HFD and stress showed shifts in 4 CAGs. Interestingly, though, 2 of the 4 were not affected by either HFD alone or by stress alone, but only when HFD and stress were combined. Males that received HFD and stress showed shifts in 3 CAGs, all of which were also affected by HFD alone (Fig. 7b, “HFD Stress” rows).

## Discussion

The prevalence of anxiety disorders in women is nearly double their prevalence in men^34^. Major depression is likewise more prevalent in women^35^. Although these gender discrepancies are well established, there is a dearth of research into their underlying causes and into potential sex-linked risk factors that could serve as therapeutic targets to eliminate gender discrepancies in neuropsychiatric disorders. One study that addressed this question found gender differences in the biological response to stress, with men showing greater endocrine responses and women showing greater autonomic responses. Interestingly, men were able to recruit the autonomic system to compensate when their endocrine stress response was suppressed, whereas women did not compensate with stronger endocrine responses when autonomic activity was suppressed^36^. Another study found that greater “anxiety sensitivity” (fear of anxiety related sensations) in women correlated statistically with the increased prevalence of anxiety and depressive disorders and might contribute to the gender discrepancies^37^. The different gonadal hormones in males and females and their differing modulatory effects on anxiety and fear have also been proposed as contributing factors to the gender discrepancies in anxiety^38^.

The results of this study suggest another possible source of gender discrepancies in the prevalence of anxiety: different gut microbiota in females and males. Twenty-eight OTUs were significantly different in female compared to male mice at baseline (Day 0), even though all mice were housed in the same facility and fed the same diet (NCD). The HFD produced significant changes in 41 OTUs in females and 32 OTUs in males by Day 81. Interestingly, however, only 10 of these significantly shifted OTUs were common to both females and males. These 10 OTUs shifted in the same direction in females and males (either increased in both or decreased in both), indicating a common response to HFD. The other 31 OTUs in females and 22 OTUs in males did not respond significantly to HFD in mice of the opposite gender.

Mice also showed gender-specific differences in the behavioral changes that occurred together with the gut microbiota changes in response to HFD. Male mice showed a significant increase in anxiety-like behaviors after 81 days on HFD, whereas females on HFD showed no such changes, although relative body weight increase was the same for females and males. These results suggest that females were more resistant than males to the anxiogenic effects of the unhealthful HFD.

When mice on NCD were subject to stress, the female and male gut microbiota again responded differently. Thirty-three OTUs in females and 40 OTUs in males changed significantly from pre-stress to post-stress (Day 81 to Day 136), but only 3 of these OTUs (OTUs 51, 157, and 37) were common to both females and males. Behavioral changes in response to stress also showed gender discrepancies. Male mice on the HDF showed reduced locomotor activity after stress compared to male mice on the NCD, whereas diet did not affect post-stress activity levels in females. It is unlikely that increased body weight alone can account for the post-stress activity decrease in HFD males, because female mice on HFD showed the same relative weight gain as males but were as active as their NCD counterparts. After normalization to eliminate the confounding effects of reduced novelty during the second exposure to the test arenas, male mice on the NCD appeared resistant to the effects of stress on activity, whereas HFD males and female mice on both diets showed decreased locomotor activity after stress.

Chronic unpredictable mild stress was previously shown to increase anxiety and alter locomotor activity in male mice^39, 40^. It also induced anhedonic behavior, but only in 50–70% of the mice^39, 40^. Anhedonia, when it occurred, was associated with depressive symptoms such as increased floating in the forced swim test. This separation of anxiety from anhedonia and other depressive symptoms caused by chronic mild stress raises an interesting question for a future study—is the gut microbiota different in the subset of mice that exhibit anhedonia and depressive symptoms compared to those that only show anxiety and altered locomotor activity?

A limitation of this study is that all mice were exposed to stress, so changes observed after completion of the stress protocol may be partially attributable to time (age). We have previously demonstrated, however, that the mouse gut microbiota is relatively stable in adult mice with no significant age-related shifts occurring between 62 and 83 weeks of age, suggesting that the differences we observed after stress are not simply attributable to age^41^. In addition, the principle coordinate analysis shown in Fig. 4a reveals that the gut microbiota of mice on NCD underwent only small shifts between days 0 and 81, again indicating that composition of the gut microbiota is relatively stable in adult mice over time, consistent with stress being the primary driver of microbiota changes observed between days 81 and 136 in this study. We also acknowledge that the chronic unpredictable mild stress protocol could introduce new microbes into the mice. To minimize the chances that this could introduce differences between groups, we ensured that identical stress conditions were applied among all the treatment groups. In addition, statistical analyses were focused on detecting differences between diet and gender treatment groups after all had been exposed to the same stress conditions, rather than comparing the same treatment group before and after stress. These measures increase confidence that the gender-specific differences observed post-stress are, in fact, attributable to stress.

When all three time points (0, 81, and 136 days) and both diets were combined into a single Random Forest modeling analysis to identify all OTUs that were changed significantly by diet or stress, the resulting heat map of female gut microbiota was striking. The heat maps of the two diets, NCD and HFD, were dramatically different from each other at Day 81, before stress. After stress, however, the NCD and HFD heat maps of female microbiota were more similar to each other. Stress caused the NCD gut microbiota to resemble the HFD microbiota—suggesting that stress and HFD can cause similar changes to the gut microbiota in females. Future studies should examine the overall health impact of stress in female mice to see if it resembles the health impact of HFD and obesity, as the microbiota shift observed herein would predict. Males, in contrast, did not show this pattern. Examination of individual OTUs supported this conclusion—in females, 8 of the same OTUs were key responders to both HFD and stress, whereas in males only one OTU responded to both HFD and stress.

A group of OTUs that increase or decrease together in response to diet and stress may do so because of metabolic interactions between members of the group. The most abundant members of such a group are not necessarily the most metabolically relevant to the host, however, so identifying co-abundance groups (CAGs) increases the odds of identifying the OTUs that are most physiologically relevant. Identification of CAGs within the combined female and males samples also revealed gender discrepancies in the gut microbiota’s response to HFD and to stress. Of the 22 CAGs identified, 7 were shifted in abundance by both HFD and stress in females and 4 were shifted by both HFD and stress in males, supporting the conclusion that stress mimicked the effects of HFD in the female more strongly than in the male gut microbiota.

Identifying the CAGs that are associated with certain behavioral patterns opens the way for future studies aimed at identifying mechanism by which specific bacteria could affect behavior. For example, in the present study HFD induced anxiety-like behaviors in male but not female mice. CAG7 and CAG21 were shifted by HFD in male but not female mice, pointing to members of these CAGs as candidate contributors to anxiety. Each candidate can now be evaluated for its genetic and metabolic potential to communicate with the central nervous system by means of autonomic, neuroendocrine, immune, or neurotransmitter systems, and top candidates can undergo subsequent mechanistic testing^42^.

In 2014, the United States National Institutes of Health (NIH) mandated sex and gender inclusion in all preclinical animal and cell culture studies funded by the agency^43^. This study illustrates the importance of that mandate, as diet and stress produced different impacts on the gut microbiota, on anxiety-like behaviors, and on locomotor activity in female and male mice.

## Electronic supplementary material

Supplementary information

## References

[1] Murphy, E. A., Velazquez, K. T. & Herbert, K. M. Influence of high-fat diet on gut microbiota: a driving force for chronic disease risk. *Current opinion in clinical nutrition and metabolic care*, doi:10.1097/mco.0000000000000209 (2015). 10.1097/MCO.0000000000000209 PMC4578152 26154278

[2] Turnbaugh PJ, Backhed F, Fulton L, Gordon JI (2008). Diet-induced obesity is linked to marked but reversible alterations in the mouse distal gut microbiome. Cell host & microbe.

[3] Ridaura VK (2013). Gut microbiota from twins discordant for obesity modulate metabolism in mice. Science.

[4] Fei N, Zhao L (2013). An opportunistic pathogen isolated from the gut of an obese human causes obesity in germfree mice. The ISME journal.

[5] Cani PD (2008). Changes in gut microbiota control metabolic endotoxemia-induced inflammation in high-fat diet-induced obesity and diabetes in mice. Diabetes.

[6] Vrieze A (2012). Transfer of intestinal microbiota from lean donors increases insulin sensitivity in individuals with metabolic syndrome. Gastroenterology.

[7] Alizai PH (2015). Presurgical assessment of bariatric patients with the Patient Health Questionnaire (PHQ)–a screening of the prevalence of psychosocial comorbidity. Health and quality of life outcomes.

[8] Mansur RB, Brietzke E, McIntyre RS (2015). Is there a “metabolic-mood syndrome”? A review of the relationship between obesity and mood disorders. Neuroscience and biobehavioral reviews.

[9] Andre, C., Dinel, A. L., Ferreira, G., Laye, S. & Castanon, N. Diet-induced obesity progressively alters cognition, anxiety-like behavior and lipopolysaccharide-induced depressive-like behavior: Focus on brain indoleamine 2,3-dioxygenase activation. *Brain, behavior, and immunity*, doi:10.1016/j.bbi.2014.03.012 (2014). 10.1016/j.bbi.2014.03.012 24681251

[10] Valleau JC, Sullivan EL (2014). The impact of leptin on perinatal development and psychopathology. Journal of chemical neuroanatomy.

[11] Castanon N, Luheshi G, Laye S (2015). Role of neuroinflammation in the emotional and cognitive alterations displayed by animal models of obesity. Frontiers in neuroscience.

[12] Sivanathan S, Thavartnam K, Arif S, Elegino T, McGowan PO (2015). Chronic high fat feeding increases anxiety-like behaviour and reduces transcript abundance of glucocorticoid signalling genes in the hippocampus of female rats. Behavioural brain research.

[13] Bruce-Keller AJ (2014). Obese-type Gut Microbiota Induce Neurobehavioral Changes in the Absence of Obesity. Biological psychiatry.

[14] Bailey MT (2014). Influence of stressor-induced nervous system activation on the intestinal microbiota and the importance for immunomodulation. Advances in experimental medicine and biology.

[15] Bailey MT (2011). Exposure to a social stressor alters the structure of the intestinal microbiota: implications for stressor-induced immunomodulation. Brain, behavior, and immunity.

[16] Bailey MT (2010). Stressor exposure disrupts commensal microbial populations in the intestines and leads to increased colonization by Citrobacter rodentium. Infection and immunity.

[17] Galley JD (2014). Exposure to a social stressor disrupts the community structure of the colonic mucosa-associated microbiota. BMC microbiology.

[18] Tarr, A. J. *et al*. The prebiotics 3′Sialyllactose and 6′Sialyllactose diminish stressor-induced anxiety-like behavior and colonic microbiota alterations: Evidence for effects on the gut–brain axis. *Brain, behavior, and immunity*, doi:10.1016/j.bbi.2015.06.025. 10.1016/j.bbi.2015.06.025 PMC4631662 26144888

[19] Chakravarty S (2014). Epigenetic regulatory mechanisms in stress-induced behavior. International review of neurobiology.

[20] Nasca C, Bigio B, Zelli D, Nicoletti F, McEwen BS (2015). Mind the gap: glucocorticoids modulate hippocampal glutamate tone underlying individual differences in stress susceptibility. Mol Psychiatry.

[21] Lin EJ (2015). Social overcrowding as a chronic stress model that increases adiposity in mice. Psychoneuroendocrinology.

[22] Gould, T., Dao, D. & Kovacsics, C. In *Mood and Anxiety Related Phenotypes in Mice* Vol. 42 *Neuromethods* (ed Todd D. Gould) Ch. 1, 1–20 (Humana Press, 2009).

[23] Komada, M., Takao, K. & Miyakawa, T. Elevated plus maze for mice. *Journal of visualized experiments: JoVE*, doi:10.3791/1088 (2008). 10.3791/1088 PMC2762911 19229173

[24] Can, A. *et al*. The mouse forced swim test. *Journal of visualized experiments: JoVE*, e3638, doi:10.3791/3638 (2012). 10.3791/3638 PMC3353513 22314943

[25] Can, A. *et al*. The tail suspension test. *Journal of visualized experiments: JoVE*, e3769, doi:10.3791/3769 (2012). 10.3791/3769 PMC3353516 22315011

[26] Godon JJ, Zumstein E, Dabert P, Habouzit F, Moletta R (1997). Molecular microbial diversity of an anaerobic digestor as determined by small-subunit rDNA sequence analysis. Applied and environmental microbiology.

[27] Edgar RC (2013). UPARSE: highly accurate OTU sequences from microbial amplicon reads. Nat Methods.

[28] Wang Q, Garrity GM, Tiedje JM, Cole JR (2007). Naive Bayesian classifier for rapid assignment of rRNA sequences into the new bacterial taxonomy. Applied and environmental microbiology.

[29] Price MN, Dehal PS, Arkin AP (2009). FastTree: computing large minimum evolution trees with profiles instead of a distance matrix. Molecular biology and evolution.

[30] Caporaso JG (2010). QIIME allows analysis of high-throughput community sequencing data. Nat Methods.

[31] Zhang, C. *et al*. Interactions between gut microbiota, host genetics and diet relevant to development of metabolic syndromes in mice. *The ISME journal* **4**, doi:10.1038/ismej.2009.112 (2010). 10.1038/ismej.2009.112 19865183

[32] Knights D, Costello EK, Knight R (2011). Supervised classification of human microbiota. FEMS microbiology reviews.

[33] Benjamini Y, Hochberg Y (1995). Controlling the False Discovery Rate - a Practical and Powerful Approach to Multiple Testing. J Roy Stat Soc B Met.

[34] McLean CP, Asnaani A, Litz BT, Hofmann SG (2011). Gender differences in anxiety disorders: prevalence, course of illness, comorbidity and burden of illness. Journal of psychiatric research.

[35] Schuch JJ, Roest AM, Nolen WA, Penninx BW, de Jonge P (2014). Gender differences in major depressive disorder: results from the Netherlands study of depression and anxiety. Journal of affective disorders.

[36] Ali N, Cooperman C, Pruessner J (2015). Sex and gender differences in the suppression of the stress systems: A potential link to anxiety and mood disorders?. Psychoneuroendocrinology.

[37] Norr AM, Albanese BJ, Allan NP, Schmidt NB (2015). Anxiety sensitivity as a mechanism for gender discrepancies in anxiety and mood symptoms. Journal of psychiatric research.

[38] Toufexis DJ, Myers KM, Davis M (2006). The effect of gonadal hormones and gender on anxiety and emotional learning. Hormones and behavior.

[39] Strekalova T, Spanagel R, Bartsch D, Henn FA, Gass P (2004). Stress-induced anhedonia in mice is associated with deficits in forced swimming and exploration. Neuropsychopharmacology: official publication of the American College of Neuropsychopharmacology.

[40] Strekalova T, Steinbusch HW (2010). Measuring behavior in mice with chronic stress depression paradigm. Progress in neuro-psychopharmacology & biological psychiatry.

[41] Zhang C (2013). Structural modulation of gut microbiota in life-long calorie-restricted mice. Nat Commun.

[42] Rieder, R., Wisniewski, P. J., Alderman, B. L. & Campbell, S. C. Microbes and mental health: A review. *Brain, behavior, and immunity*, doi:10.1016/j.bbi.2017.01.016 (2017). 10.1016/j.bbi.2017.01.016 28131791

[43] Clayton JA, Collins FS (2014). Policy: NIH to balance sex in cell and animal studies. Nature.

