# Supplementary material for: Gender-based differences in host behavior and gut microbiota composition in response to high fat diet and stress in a mouse model
Source: Sci Rep. 2017 Sep 7;7:10776. doi: 10.1038/s41598-017-11069-4 (PMC5589737; doi:10.1038/s41598-017-11069-4)
Supplement: Supplementary file 1 — Supplementary information [file 41598_2017_11069_MOESM1_ESM.pdf]

Gender-based differences in host behavior and gut microbiota composition in response to high fat diet and stress in a mouse model

Laura C. Bridgewater[ab]*, Chenhong Zhang[b]*, Yanqiu Wu[b], Weiwei Hu[c], Qianpeng Zhang[b], Jing Wang[b], Shengtian Li[c], Liping Zhao[b§]

[a] *Department of Microbiology and Molecular Biology, Brigham Young University, Provo, Utah, USA*

[b] *State Key Laboratory of Microbial Metabolism, School of Life Sciences and Biotechnology, Shanghai Jiao Tong University, Shanghai, P. R. China*

[c] *Bio-X Institutes, Shanghai Jiao Tong University, Shanghai, P. R. China*

*These authors contributed equally to this work

§ Corresponding author:

Dr. Liping Zhao

Email: lpzhao@sjtu.edu.cn

Supplementary Information

Table 1 The high-quality reads of samples.

Day 0							
Female				Male			
NCD	high-quality reads	HFD	high-quality reads	NCD	high-quality reads	HFD	high-quality reads
A01	9100	B01	7624	C01	8657	D01	9676
A02	4775	B02	6357	C02	5343	D02	818
A03	7535	B03	7343	C03	5112	D03	8065
A04	4933	B04	5647	C04	7023	D04	7040
A05	4080	B05	5992	C05	7104	D05	6454
A06	7286	B06	6133	C06	4819	D06	5768
A07	3898	B07	5407	C07	9038	D07	10027
A08	4877	B08	5786	C08	7249	D08	9179
A09	4424	B09	6207	C09	5808	D09	8677
A10	6485	B10	7201	C10	6221	D10	6500
A11	8291	B11	5519	C11	5298	D11	6011
A12	5362	B12	8081	C12	8337	D12	4330
A13	6311	B13	7795	C13	7581	D13	9728
A14	3327	B14	7132	C14	7718	D14	6013
A15	6774	B15	5695	C15	5869	D15	5972
A16	5250	B16	4154	C16	7070	D16	6198
A17	8711	B17	6830			D17	6951
Day 81							
Female				Male			
NCD	high-quality reads	HFD	high-quality reads	NCD	high-quality reads	HFD	high-quality reads
A01	6538	B01	7109	C01	9068	D01	10897
A02	4497	B02	5644	C02	6857	D02	10203
A03	8097	B03	4374	C03	6506	D03	9054
A04	8289	B04	8358	C04	6751	D04	9437
A05	7452	B05	6362	C05	10449	D05	10160
A06	8315	B06	6972	C06	6603	D06	9215
A07	4629	B07	7310	C07	9236	D07	13464
A08	9872	B08	7876	C08	7658	D08	6838
A09	9005	B10	7291	C09	9916	D09	6642
A10	6615	B11	5573	C10	10374	D10	9865
A12	6477	B12	6914	C11	8961	D11	9061
A13	5674	B13	9143	C12	7163	D12	9619
A14	8171	B14	8519	C13	4892	D13	8618
A15	5543	B16	11691	C14	7282	D14	11200
A16	7416	B17	10226	C15	6157	D15	6887
A17	9431			C16	7996	D16	11332
						D17	9387
Day 136							
Female				Male			
NCD	high-quality reads	HFD	high-quality reads	NCD	high-quality reads	HFD	high-quality reads
A03	9751	B01	5010	C02	6913	D01	7639
A04	7148	B02	11257	C03	5672	D02	5565
A05	9425	B03	8697	C04	5291	D03	4482
A06	6988	B05	5624	C05	8212	D04	6343
A07	7589	B07	8435	C06	7863	D05	8338
A08	8000	B09	7187	C07	8296	D06	11069
A09	9283	B10	6151	C08	5791	D07	6009
A10	11876	B11	7849	C09	6829	D08	5665
A11	10904	B12	4856	C10	7164	D10	8289
A12	7534	B13	5838	C11	8770	D11	7860
A13	8316	B14	9288	C12	8713	D13	9042
A14	8702	B15	8256	C13	8813	D14	4466
A15	8538	B16	8576	C14	7567	D15	7548
A16	6757	B17	5123	C15	2421	D16	8081
A17	9014			C16	7037	D17	5917

Supplementary Figure 1

Normal chow diet (NCD)

Formula

Product #D12450B		gm%	kcal%
Protein		19.2	20
Carbohydrate		67.3	70
Fat		4.3	10
	Total		100
	kcal/gm	3.85	

Ingredient	gm	kcal
Casein, 30 Mesh	200	800
L-Cystine	3	12
Corn Starch	315	1260
Maltodextrin 10	35	140
Sucrose	350	1400
Cellulose, BW200	50	0
Soybean Oil	25	225
Lard*	20	180
Mineral Mix S10026	10	0
DiCalcium Phosphate	13	0
Calcium Carbonate	5.5	0
Potassium Citrate, 1 H2O	16.5	0
Vitamin Mix V10001	10	40
Choline Bitartrate	2	0
FD&C Yellow Dye #5	0.05	0
**Total**	1055.05	4057

Formulated by E. A. Ulman, Ph.D., Research Diets, Inc., 8/26/98 and 3/11/99.

*Typical analysis of cholesterol in lard = 0.72 mg/gram.
Cholesterol (mg)/4057 kcal = 14.4
Cholesterol (mg)/kg = 13.6

High fat diet (HFD)

Formula

Product #D12492		gm%	kcal%
Protein		26.2	20
Carbohydrate		26.3	20
Fat		34.9	60
	Total		100
	kcal/gm	5.24	

Ingredient	gm	kcal
Casein, 30 Mesh	200	800
L-Cystine	3	12
Corn Starch	0	0
Maltodextrin 10	125	500
Sucrose	68.8	275.2
Cellulose, BW200	50	0
Soybean Oil	25	225
Lard*	245	2205
Mineral Mix S10026	10	0
DiCalcium Phosphate	13	0
Calcium Carbonate	5.5	0
Potassium Citrate, 1 H2O	16.5	0
Vitamin Mix V10001	10	40
Choline Bitartrate	2	0
FD&C Blue Dye #1	0.05	0
**Total**	773.85	4057

Formulated by E. A. Ulman, Ph.D., Research Diets, Inc., 8/26/98 and 3/11/99.

*Typical analysis of cholesterol in lard = 0.72 mg/gram.
Cholesterol (mg)/4057 kcal = 216.4
Cholesterol (mg)/kg = 279.6

Supplementary Figure 1 | Formulas of the normal chow diet (NCD) and high fat diet (HFD) used in this study, reproduced from product data sheets provided by Research Diets Inc.

Supplementary Figure 2

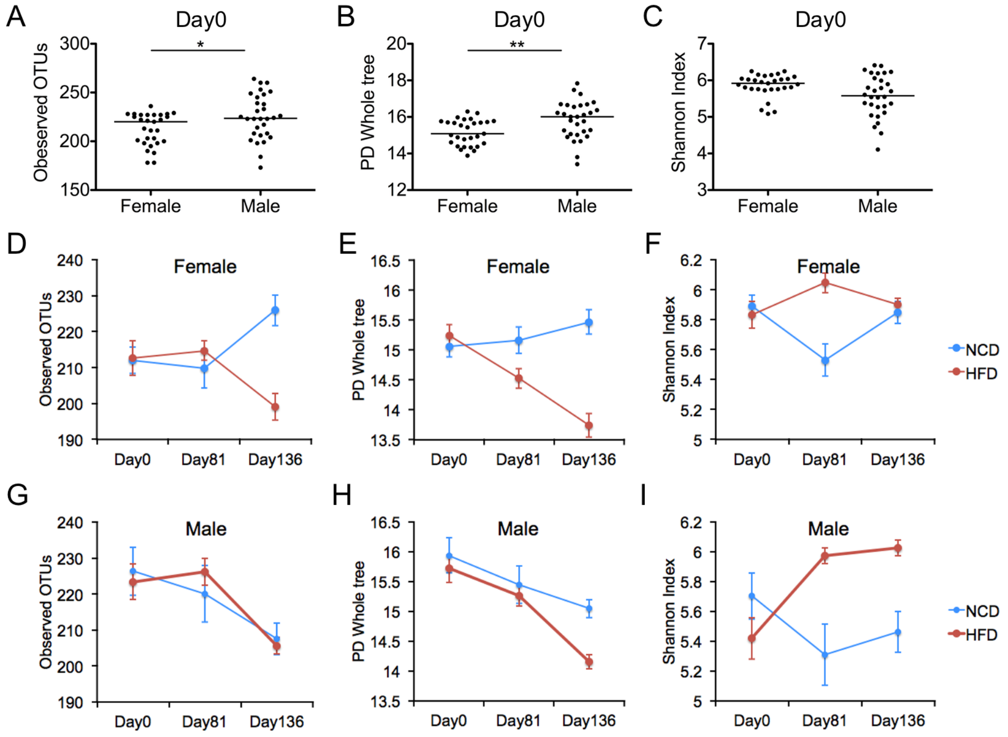

Supplementary Figure 2 | The alpha-diversity of samples were calculated with observed OTUs, Faith's phylogenetic diversity (PD Whole tree) and the Shannon index.

Supplementary Figure 3

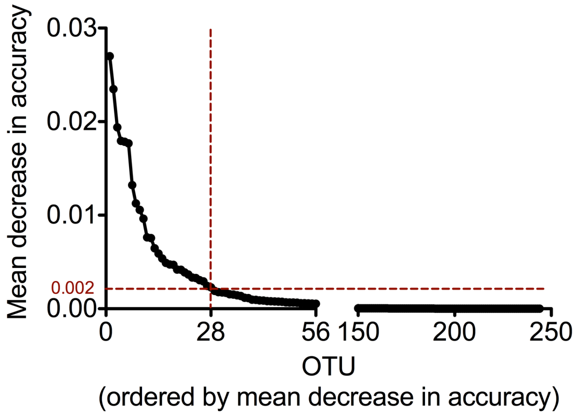

Supplementary Figure 3 | key OTUs were identified by applying Random Forest classification for different sex on Day0, ranked in descending order of their feature accuracy of the models.

Supplementary Figure 4

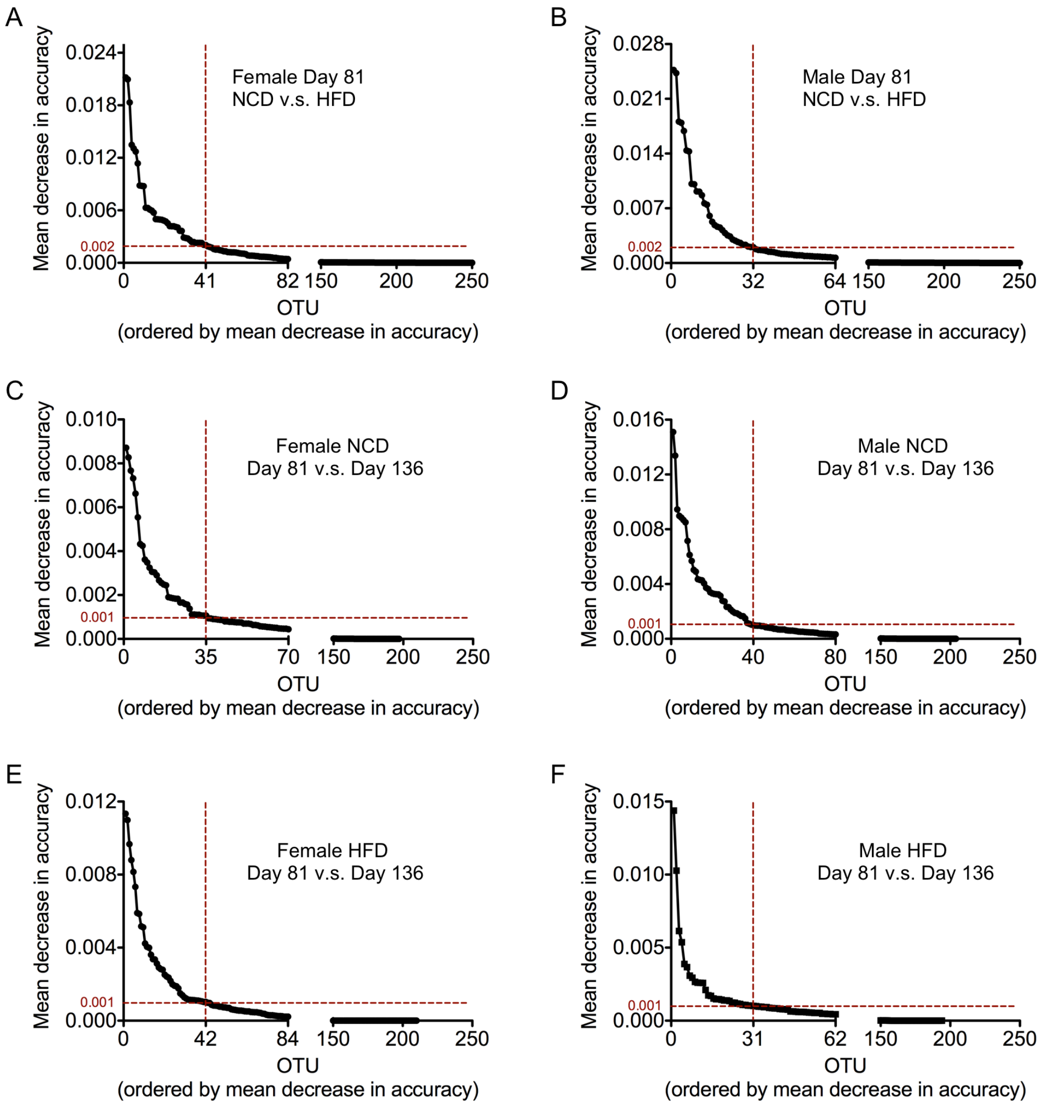

Supplementary Figure 4 | key OTUs were identified by applying Random Forest classification for (A) different diet of female, (B) different diet of male, (C) stress of female on NCD, (D) stress of male on NCD, (E) stress of female on HFD and (F) stress of male on HFD, ranked in descending order of their feature accuracy of the models.

Supplementary Figure 5

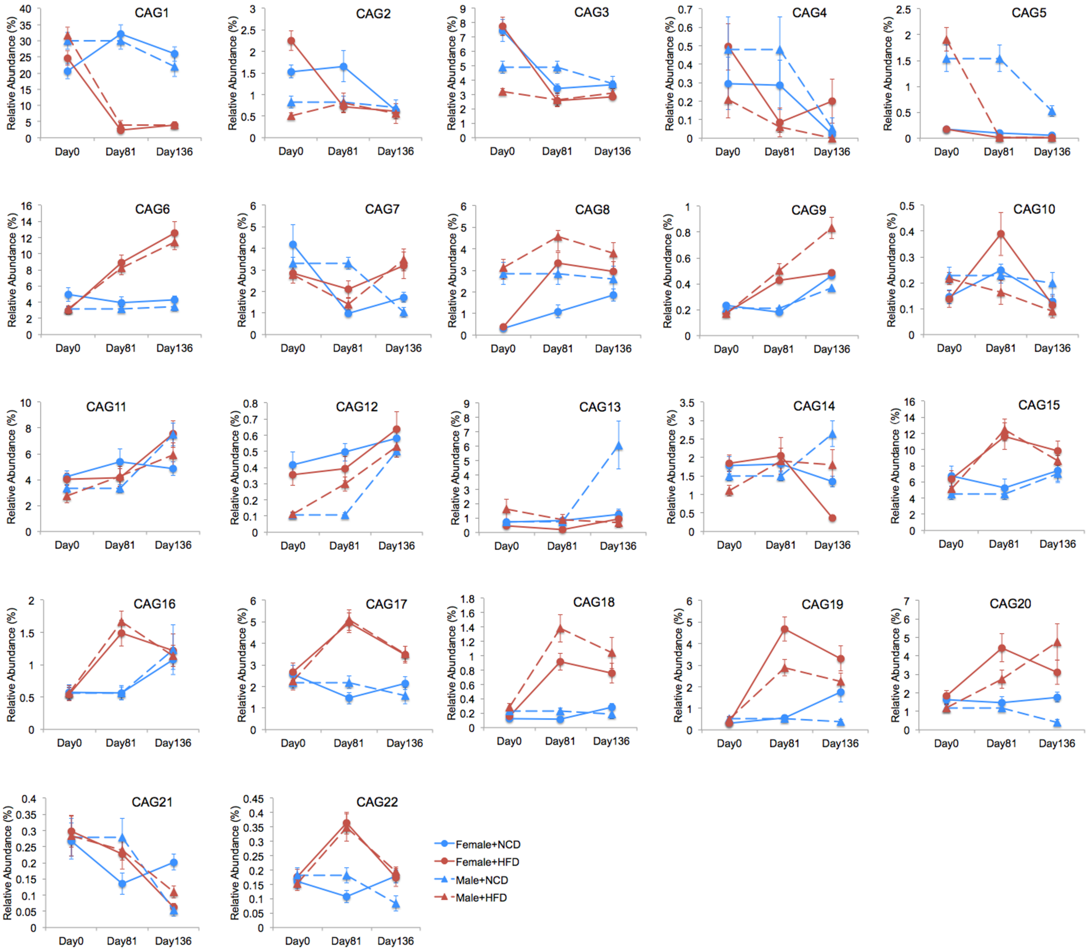

Supplementary Figure 5 | Group level abundance shifts of CAGs during intervention.
